# Beyond the medical file: A scoping review on patients’ perspectives on depression treatment in primary care

**DOI:** 10.1371/journal.pone.0293713

**Published:** 2025-06-05

**Authors:** Katharina Biersack, Heribert Sattel, Petra Schönweger, Lea Kaspar, Nadine Lehnen, Jochen Gensichen, Peter Henningsen, for the POKAL group

**Affiliations:** 1 Department of Psychosomatic Medicine and Psychotherapy, University Hospital of Technical University of Munich, Munich, Germany; 2 Faculty of Medicine, Chair of Public Health and Health Service Research, Institute of Medical Information Processing, Biometry and Epidemiology (IBE), LMU Munich, Munich, Germany; 3 Pettenkofer School of Public Health, Munich, Germany; 4 Institute of General Practice and Family Medicine, University Hospital of Ludwig-Maximilian-University Munich, Munich, Germany; Mahidol University, THAILAND

## Abstract

**Objectives:**

Depressive disorders are common in the primary care setting. Despite its high prevalence, depression treatment in primary care is less guideline-oriented compared to specialized settings, which often makes it less efficient. Current research has focused on explanations on the practitioner’s side but has neglected the patient’s perspective and its effect on treatment largely.

**Methods:**

We conducted a scoping review on the electronic databases Medline and Psycinfo. Eligible publications contained information of the patients’ perspective on depression treatment in primary care in OECD member states. Publications until August 2nd 2023 were considered.

**Results:**

After the removal of duplicates, the search yielded 14.059 articles, of which 232 were included. Current literature focuses on behavioral and obvious measures like satisfaction, and on patient-sided barriers and facilitators to adherence. Other treatment-related behaviors are less researched. Patients with undiagnosed depression often report exclusively or mainly physical symptoms in general practice.

**Conclusion:**

This review provides a comprehensive framework for the concept. Research on barriers and facilitators to depression treatment in primary care is still inconclusive. Educating patients and addressing stigmatizing beliefs are promising targets to promote the seeking out, initiation of, and adherence to treatment. Being aware of a hidden depression when somatic symptoms are present, can help to detect more cases.

**Registration:**

This review is registered via OSF (https://osf.io/p9rnc).

## 1. Introduction

Depressive disorders are very common in general practices and are often reason for disability and absenteeism from work [1,2]. Undetected and inadequately treated cases of depression are a burden to patients, the healthcare system, and society [3]. Still, depression is often undiagnosed and, even if diagnosed, untreated or not treated according to guidelines [4]. To this day, the practitioner’s view is regularly researched while the patient’s perspective on the reasons for these gaps in diagnosis and treatment are not completely clear.

Clinical practice guidelines (CPGs) have long been used to bridge the gap between best practice and real-world primary care. While most high and middle-income countries have national guidelines for depression treatment [5], many guidelines are not adequately implemented in primary care settings [6].

This can be attributed, in part, to guidelines themselves, as only a few provide effective implementation guidance. For example, the German national CPG for depression treatment mentions “implementation” only twice in its 257 pages with no clear guidance on the topic [7]. A systematic review concluded that up to now guideline implementation is inadequately planned, reported, and measured globally [8]. Barriers to CPG usage in primary care, such as lack of education or limited consultation time, have been a subject of interest and research among clinicians and stakeholders [9]. A recent simulation study revealed that primary care professionals would require more than 26 hours per day to implement, use, and document according to all current CPGs. 1.6 hours would be solely dedicated to mood disorders [10].

To improve evidence-based treatment for depressed patients in primary care, various reviews highlight systemic problems requiring large-scale policies [8,9,11]. However, it takes considerable time before these policies effectively enhance patient care. In the meantime, research should focus on identifying gaps and exploring alternative approaches.

One significant gap lies in understanding of the patients’ perspective on treatment. Patient-reported and -relevant outcomes are a topic of growing interest as patient-centered care is required to decide what is meaningful and valuable to the individual patient [12]. Patients’ perspectives, their values and preferences should be considered in the clinical decision-making process [13]. Up to now, patient-reported outcomes often consist in symptom-related questionnaires but do neglect patients’ point of view on domains such as satisfaction, expectations and contextual information [14]. Moreover, patient-relevant outcomes are underrepresented in the current literature despite being important in the treatment [15].

But what about patients’ point of view? What does it consist of and how has it been studied? Currently, there are no frameworks or reviews available on how patients, as key stakeholders, perceive their care. Just recently, a review emphasized the need for increased patient participation in guideline development and to date only few guidelines involve patients in the process [5]. Still, there is no definition of this concept or comprehensive overview on how it can be studied and compared.

Could patients’ perspectives serve as the missing link between evidence and its implementation into practice offering a whole range of new intervention strategies to improve treatment outcomes? Patients’ experiences of depression treatment in primary care, barriers to help-seeking, and factors affecting their understanding of and engagement in treatment are vital for effective treatment. With this scoping review we provide a systematic overview to help guide future research defining ‘patients’ perspective on depression treatment in primary care’ based on studied barriers and facilitators to patients’ side of depression care.

## 2. Aim

This scoping review aimed at defining the concept of ‘patients’ perspectives on primary care’ to provide a comprehensive framework of related research topics and methods.

The primary aim was to identify the scientific methods and concepts related to patients’ perspectives on depression treatment in primary care.

Secondly, within the framework, we aimed to identify already researched barriers and facilitators towards evidence-based treatment of depression in primary care.

## 3. Methods

### 3.1. Search strategy

We conducted a scoping review employing the electronic databases of Medline and Psycinfo. The study was in accordance with the JBI Manual for Evidence Synthesis [16] and guided by the PCC (population, context, concept) [16] -framework (Table 1). The protocol was registered with Open Science Framework (https://osf.io/p9rnc). For the applied search terms see appendix. Search terms were based on the PCC framework (See below).

**Table 1:** PCC-Framework.

Population	Adult currently clinically depressed patients in OECD member states
Context	Primary care as defined by the authors, all available studies
Concept	Patients’ perspectives on depression care, i.e., their behavior, mindset, and experience

### 3.2. Inclusion and exclusion criteria

We included publications available in English, German, Spanish or French as we felt confident enough to judge and extract information in these languages. The inclusion criteria were: adults with a current clinical diagnosis of depression, treatment in the primary care context within the OECD states, and data on the patients’ perspective (see below for definition).

To avoid missing relevant insights, we included all papers up August 2^nd^, 2023, recognizing that older studies might address topics neglected in recent years. Given the lack of an existing framework on this concept, a comprehensive approach seemed essential to provide a thorough overview.

The exclusion criteria were: children, no clear definition of depression or other mental health issues apart from depression, treatment in specialized care or treatment in hospital, set in Non-OECD states and no data on the patients’ perspective.

Further details on inclusion and exclusion criteria are given in detail within the descriptions of population, context and concept.

#### 3.2.1. Population.

We included studies from OECD member states with adult persons with depression, regardless of how it was diagnosed as long as the term ‘depression’ was used and the diagnostic method was described. We defined ‘depression’ as an up-to-date, ICD or DSM based diagnosis in the protocol but changed the definition to not to be too exclusive.

It proved too exclusive to include only publications with a clear stated definition of the diagnosis of depression, as many publications included subjects diagnosed by their primary care practitioner (PCP) without further specification. To tackle our goal to provide a scope to the field, we decided to include all studies that clearly and comprehensively stated how they diagnosed depression.

If the study included both depressed and dysthymic patients, it was still included for reasons of practicality. For the same reason, we included studies conducted in patients who were selected because of an antidepressant prescription if the authors stated that the prescription was for the treatment of depression and not for some other disorder, e.g., anxiety. We also included publications in which the diagnosis was given by the PCP, even if their approach was not clearly stated, defining ‘diagnosis by PCP’ as a diagnostic method if no other measures were specified.

#### 3.2.2. Context.

We selected papers that referred to the setting as ‘primary care’ and defined the setting. Patients did not necessarily have to be recruited in primary care but had to give information about their diagnosis or treatment in that context. We included all ambulatory settings meeting these criteria.

#### 3.2.3. Concept.

We decided to include papers that contained information from the patients’ own account of and spontaneous behavior towards their care. The articles had to relate not just to symptomatology but also to the care and depression management itself. To define the concept and thus the search strategy we carried out extensive preliminary research for relevant publications. From these, we extracted topics for our concept. We then grouped the emerging topics into the categories ‘mindset’, ‘experience’, and ‘behavior’. For definition see below.

The three main conceptual dimensions of the search strategy were 1.) patients’ mindset (including thoughts and feelings towards depression care, their mindset before or during care), 2.) patients’ experience of care (e.g., qualitative accounts on experience or even binarily given satisfaction),and 3.) patients’ behavior (e.g., taking medication or using health care facilities). We excluded publications limited to psychopathological outcomes as these were not part of our definition of ‘patients’ perspective on care’.

***3.2.3.1. Mindset.*** We sought to include patients’ own concept of depression and treatment, and its importance for the beginning and continuity of care. After scoping the literature, we identified various pre-existing concepts describing aspects of the mind related to action, e.g., ‘attitude’, ‘belief’, ‘knowledge’, or ‘expectation’. These different terms and concepts together were summarized under ‘mindset’, as we wanted the framework to be able to include further topics, thus the term had to be broad enough. We defined ‘mindset’ as all mental states, e.g., attitudes, prior knowledge, beliefs, preferences, related to the patient-sided uptake and maintenance of depression treatment in primary care.

***3.2.3.2. Experience.*** To conceptualize terms describing the patients’ point of view while in treatment, we used the term ‘Experience’. We included studies in which patients were a distinguishable group and their information added to the concept. ‘Experience’ was defined as patients’ account on steps of their actual treatment as well as their judgement given as, e.g., satisfaction.

***3.2.3.3.*** ***Behavior***. We included behavior in our definition of the concept because it is directly related to motivation and can indicate potential barriers and facilitators. It is also straightforward to detect and to describe from a research point of view. We defined ‘behavior’ as reports of patient-sided treatment-related behavior given by patients themselves, information given by their primary care practitioners as well as information on patient behavior collected and given by a third party, e.g., researchers.

Therefore, the search terms ‘consultation’ or ‘help seeking’ were included. To look for patterns of symptom report, we included studies that used open-ended measures to describe the complaints with which patients proactively presented, e.g., the reason for consultation the general practitioner (GP) noted, as opposed to being specifically asked for depressive symptoms. We excluded studies in which symptoms were given via broad psychometric testing because answers are pre-empted by the questionnaires. We only included symptom reports if symptoms were given proactively, e.g., as reason for the doctors’ visit. We used the search terms ‘symptoms’ and ‘complaints’ for that purpose.

***3.2.3.4. Barriers and facilitators.*** We defined ‘barriers and facilitators to depression treatment in primary care from the patients’ point of view’ as factors that impede or help with therapy-related patient behaviors.

Therefore, we included the terms ‘barrier’ or ‘facilitator’ in our search. With the before-mentioned search strategy we felt confident to also identify all other articles studying barriers and facilitators that did not use these terms but conducted and presented their investigation in a way that allowed us to include their data by means of our definition.

### 3.3. Search terms

We used the PCC framework and our definition of the three categories to build our search strategy. While working on it we had already identified some of the terms we wanted to search for. Within several group meetings and searches on Medline we decided on the search terms that can be found in the Appendix. We used Medline’s Mesh-terms whenever applicable and plain words and phrases when the respective Mesh-term seemed too exclusive or was not existent, e.g., in the case of “expectations”. In the Appendix, pretests for all the applied Mesh-terms and iterations done on Medline before running our search on both Medline and PsycINFO are available.

The complete search was built according to the PCC structure, i.e., firstly search terms for the population, secondly, linked with an ‘AND’, the terms for the context and thirdly, again linked with an ‘AND’, the search terms related to the concept. Therefore, all primary search results were linked to terms related to the population, the context, and the concept we were looking for.

### 3.4. Review and synthesis process

We evaluated every step of the review process within our research group following a mixed, deductive and inductive, approach. After the pre-scoping, we decided on the dimensions, i.e., ‘mindset’, ‘experience’, and ‘behavior’ which helped build and adapt the research strategy.

For the title and abstract screening, the Rayyan software was employed. The screening was conducted by three independent raters who continuously evaluated the adherence to inclusion and exclusion criteria and adapted them when needed. All publications set in the wrong context (e.g., inpatient care), reporting on the wrong concept (all outcomes not related to patients Mindset, experience, or behavior) or the wrong population (e.g., children or patients not suffering from depression) were excluded if this was apparent from reading the abstract. Case studies and publications not offering primary information were also excluded. One hundred abstracts were rated by all three to check and guarantee consistency in criteria and their application to the data. Abstract titles and screening results are available via OSF (https://osf.io/t7562/). After inclusion, we extracted and grouped arising topics within domains and dimensions inductively.

## 4. Results

### 4.1. Search results

**Fig 1:**
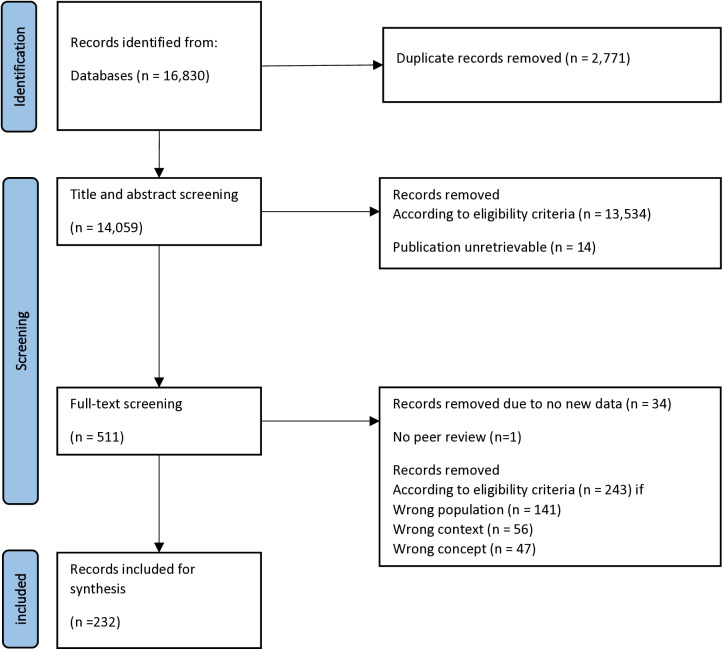
Flow diagram describing search strategy and results.

The conducted search of the databases identified 16,830 results. After the removal of 2,771 duplicates, we included 14,059 studies in the screening of title and abstract. Following the mentioned PCC-scheme, we excluded 13,534 abstracts. Of the remaining 525 studies, 14 papers could not be retrieved even after directly contacting authors and libraries and could therefore not be considered for further review. In the full text screening, we excluded 34 studies because they did not offer new data, 1 study because it lacked peer-review, 141 studies due to wrong population, 56 because of wrong context and 47 because of wrong concept. We excluded several qualitative studies on the grounds that they refused to define depression because it contradicted their approach. We included 232 studies in our data synthesis. The results are presented in a Preferred Reporting Items for Systematic Reviews and Meta-analyses for Scoping Reviews (PRISMA-ScR) flow diagram [17]. See Fig 1.

### 4.2. Studies’ characteristics and methodology

Within included articles 41%(n=95) were conducted in the US, 24% (n=54) in EU countries, 25% (n=57) in the UK, and 6% (n=13) in Australia. The remaining studies were conducted in Canada (n=3), Israel (n=3), Japan (n=2), New Zealand (n=2) and Norway (n=1). Two studies included data from more than one country [18,19].

**Fig 2:**
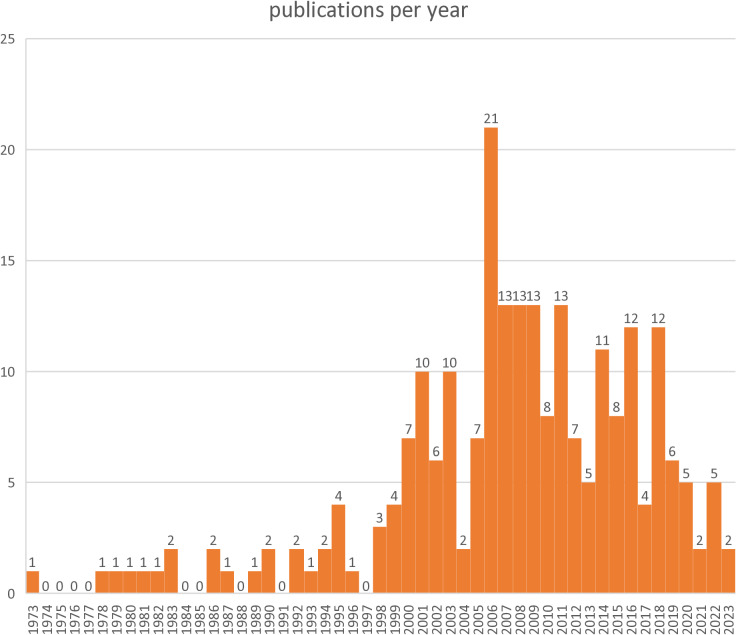
Temporal distribution of included publications.

Sixty-three percent (n=146) of the included publications used exclusively quantitative measures for outcomes of interest, while 37% (n=86) used a qualitative or mixed methods approach. For the distribution by year of publication see Fig 2.

### 4.3. Population

The biggest share of publications (38.8%) used self-rating scales to define depression and identify subjects, most studies used the *patient health questionnaire* (PHQ) PHQ-9, PHQ-8, or PHQ-2. Twenty-six percent defined ‘depressed patient’ as diagnosed by their primary care practitioner (PCP). Other publications used the prescription of antidepressants for depression, patient chart, patient history, or gave details on used checklists for diagnosis as shown in Table 2.

**Table 2:** Description of depression diagnosis in included publications. Total numbers and percentages.

Concept of depressed patient	Number of publications	%
Diagnosis by PCP	61	26.29
Diagnostic interview	47	20.26
Self-rating	46	19.83
PHQ	44	18.96
Prescription of an antidepressant	15	6.46
Chart	9	3.88
Patient history	7	3.02
Checklist	2	0.86
Diagnosis by Author	1	0.43

### 4.4. Context

Table 3 gives details on used terms in the included publications. The most used term for the primary care setting was ‘general practice’ and ‘general practitioner’ (44%). Less used but trending in more recent publications was ‘collaborative care’ (2%).

**Table 3:** Description of primary care context in included publications. Total numbers and percentages.

Concept of primary care	Number of publications	%
General practice	96	41.38
Primary care clinic	33	14.22
Primary care practice	19	8.19
Health center	11	4.74
Family practice	10	4.31
Veteran care	8	3.45
Collaborative care	1	0.43
Aged-care facility	1	0.43
Family medicine clinic	6	2.59
Other	10	4.31
Primary care	37	15.95

### 4.5. Patients’ perspectives on depression treatment in primary care

#### 4.5.1. Identified topics and domains.

In an inductive process, we identified relevant domains for the concept. The premeditated superordinate categories (dimensions), i.e., mindset, experience, and behavior, served well as a framework for the found research topics. See Table 4 for the grouped topics and domains as well as the number of their occurrences and their descriptions among the included publications. See appendix 3 for all included publications as well as extracted topics.

**Table 4:** Patients’ perspectives on depression treatment in primary care: Number of occurrences of named topics, grouped into domains within the premeditated framework of dimensions, i.e., ‘mindset’, ‘experience’ and ‘behavior’.

Patients‘ perspectives: Domains	Number of publications on the topic	Definition	Topic
Dimension: Mindset	118	Preconceived ideas, knowledge, beliefs, attitudes etc. on depression treatment in primary care.	
1. Attitude to care	29	Views on (future or possible) care, not based on experience.	Attitude to medication [20–22] Attitude to care [23] Drug attitude [24] Attitude to psychotherapy [25] Attitude to antidepressants [26] Attitudes towards discussing suicide [27] Attitude to depression and its treatment [28] Attitude to drugs [29–32] Acceptability of medication [33] Acceptability [34] Acceptability of treatment [35] Preconceptions about treatment [36] Beliefs about medication [37–40] Beliefs about psychotherapy [41] Views regarding antidepressant medication [42] Acceptability of antidepressants [43] Attitude to treatment [44–46] Views on medication [47] Medication aversion [48]
2. Concept of depression	38	Ideas and values concerning the aetiology and qualities of depression.	Health beliefs [31,35,43] Attitudes concerning depression [49] Understanding of depression [50–53] Understanding the persistence of depression [54] Understanding postnatal depression [55] Conceptualization of depression [56] Illness model [57,58] A faith-based model of depression [59] Problem formulation [60] Beliefs about depression [61–63] Belief about the cause of depression [64] Beliefs about illness [65] Source and course of depression [66] Perceived causes [67] Perceived triggers for depression [68] Perceived cause of depression [69,70] Causes of depression [71] Links between coronary heart disease and depression [72] Name of problem [70] Causal attribution [73,74] Health locus of control [75,76] Symptom interpretation [77] Attribution style [78] Concept of depression [79] Illness perception [80,81] Illness interpretation [82] Attitude to depression [28]
3. Stigma	12	Felt and reported stigma of depression.	Stigma [41,67,83–88] Perceived stigma [69] Prejudice and discrimination [89] Stigma of psychological help [90] Belief that depression is stigmatizing [48]
4. Entitlement to time	1	Feeling of entitlement to time towards GP.	Entitlement to time [91]
5. Confidence	3	Feeling confident about handling treatment or symptoms.	Confidence in own ability [92] Confidence in managing side effects [21] Controllability [57]
6. Treatment expectations	6	Prognosis of aspects of depression care.	Expectations [36,93] Expectations of depression care [56] Expectations of online CBT [94] Concerns what others might think [95] Fear of psychiatric or counselling referral [48]
7. Knowledge	3	Preliminary knowledge on depression and its treatment.	Knowledge [96] Knowledge on depression [86] Literacy for depression [89]
8. Treatment preference	26	Preferences towards aspects of treatment.	Preference [25,47,93,97–109] Desires towards depression treatment [110] Preference for involvement [76] Preference for decision-making [111] Preference for treatment [112,113] Treatment preference [83,114,115] Desire for emotional support [90] Discomfort talking about personal issues with someone unknown [95]
Dimension: Experience	134	Aspects of patients’ experience of depression treatment in primary care.	
1. Satisfaction	18	Being content with aspects of depression treatment.	Satisfaction [73,114,116–124] Satisfaction with decision [96] Satisfaction with care [35,106,125,126] Satisfaction with collaborative care management for depression [127] Satisfaction with telehealth use [128]
2. Receiving information	7	Being informed or educated about depression by GP.	Receiving information about antidepressants [129] Information given [52] Patient education [37] Being educated [130–133]
3. Reported relationship	22	Aspects of doctor-patient-relationship rated from the patient’s side.	Relationship [30,116,119,121,123,134–136] Unsure how to introduce topic [48] Role of relationship [38] Doctor-patient-communication [20] Therapeutic alliance [73] Report of provider behavior [83] Opinions of care managers [127] Ascribed role of the GP [137] Being asked [88,138] Maintaining face in medical consultations [139] Experience of empathy [140] Trust in PCP [141] Being empowered [131] Perceived norm (‘my GP thinks that I need to take medication’) [31]
4. Acceptance of diagnosis and care	15	Agreement with diagnosis and treatment course as proposed by GP.	Acceptance of care [142] Insight [37] Agreement with GP on problem [60] Acceptability [36] Acceptance of diagnosis [143,144] Acceptance of diagnosis and treatment [73] Self-identification as having a mental illness [89] Perceived need for care [89,145–147] Agreement with treatment [31,32,148] Agreement with depression label [83]
5. Reported quality of care	9	Rating the quality and judging the importance of different aspects of depression care.	Quality rating [131] Quality of care indicators [119,121] Quality of care [149] Care rating [150] Importance of different aspects of care [23] Perceived improvement factors [151] Goals for the management of depression [143] Attributing recovery [152]
6. Experience of depression	8	Experience of depression and its symptoms.	Experience of depression [71,88,134,153] Experience with depressive symptoms [154] Experience of recurring depression [155] Experience of coronary heart disease and depression [72] Self-perception of depression [156]
7. Experience of treatment	49	Other aspects of the experience of undergoing depression treatment in primary care.	Experience of online CBT [94] Experience of visits to primary care to mental health [157] Experience of depression care [56] Experience of care [123,158–161] Experience of computerized cognitive behavioral therapy [162] Experience of primary care – related mental health care [106] Experience of patient centred care [150] Patient-centredness of care [35] Advantages of telehealth use [128] Experience of treatment [36,44,45,73,122,149,152,163–167] Experience of medication [38] Experience of taking medication [129] Attitude to treatment [98] Attitudes towards care management [127] Report of received treatment [145] Experience of seeking help [69,70,168] Views on depression treatment [49,54] Views on long term use of antidepressants [51] Views on screening and treatment [144] Views on treatment [169] Patients’ perspectives on antidepressant treatment [170] Patients’ perspectives on the use of the Montgomery-Asberg depression rating scale self-assessment version in primary care [171] Patients’ experiences of using the PHQ-9 in primary care consultations [172] Opinions on treatment [68] Perspective on collaborative care [173] Experience of collaborative care [174] Views on collaborative care for depression [175] Received treatment [83] Experience of being asked about suicidality [176] Being told to stop by GP [30] Experience of disclosure [177]
8. Participation in treatment decisions	6	Being knowingly part of decisions regarding depression treatment.	Shared decision-making [178] Participation in treatment decisions [52,179] Involvement in decision making [180] Awareness of care [123] Patient-centredness of care [35]
Dimension: Behavior	158	Patients’ reported or observed behavior related to depression treatment in primary care.	
1. Health service use	45	Quantity of visits to or consultations at GP’s office.	Health care utilization [142,181–183] Health service use [184–187] Service use [19] Health service utilization [188] Health care use [189] Health care resource use [124] Number of consultations [190] Consultation rate [191] Attendance frequency [192,193] Consultation frequency [194–199] Consultation pattern [200] Number of consultations [116,145] Number of clinic visits [158] Number of visits to GP [201] Over-attendance [202] Access to primary care [203] Contact with GP [204,205] Visiting frequency [206] Visits to FD [207] Visits to GP [32,74,141,208] Mental health support use [209] Self-initiated consultations [210] Visiting health care provider [120] Number of calls [211] Medical utilization [212] Frequent attendance [213] Attendance [214,215]
2. Presenting complaints	14	Given reasons for consultation while seeking help for depression in primary care.	Complaints [18] Chief complaint [67,70,194,216] Reported complaints [87] Presenting complaints [206,217–219] Presenting symptoms [220] Reason for visit [221] Clinical presentation [222] Mentions of symptoms [223]
3. Coping	21	Patients’ strategies and characteristics towards improving mental health.	Self-management [21,100,127,224] Coping [57,225] Coping strategies [226,227] Lifestyle choices [155] Self-efficacy [108,122] Self-chosen therapies [228] Use of complementary and alternative medicine [229] Patient activation [230] Resilience [231] Patterns of mobile app use among patients with depressive symptoms [232] Illness behavior [186] Self-help [187] Empowerment [215] Relief seeking style [233] Self-help strategies [92]
4. Adherence	39	Continuing a subscribed drug or other treatment as advised by GP.	Adherence [21,24,28,29,37,39,43,57,93,111,122,130–132,149,160,166,198,234–237] Treatment adherence [179] Adherence to treatment [32,238] Adherence to antidepressants [129] Adherence to program [124] Compliance [20,30,239] Compliance to antidepressants [148] Discontinuation of antidepressants [133,240] Stopping medication [155] Medication concordance [38] Filing prescriptions [204] Completion of online CBT [94] Continuation of antidepressant treatment [31] Care engagement [83]
5. Help seeking	12	Consulting GP about depression.	Help seeking [44–46,67,88,89,166,201] Accessing help [165] Uptake of GP referral [90] Time to presentation [241] Willingness to seek help [141]
6. Initiation of treatment	14	Starting a subscribed treatment (drug or other).	Filing prescriptions [41,204] Initiation [52,69,93,95,166] Initiation of antidepressant treatment [31] Initiation of treatment [32,242,243] Taking up referral to behavioral health [135] Initiation of psychotherapy [41] Treatment initiation [160]
7. Disclosure	10	Opening up about depressive symptoms or aspects of depression treatment in consultation with GP.	Symptom disclosure [244] Disclosure of symptoms [55] Disclosure [48,177] Declaration of symptoms [245] Informing physician [30] Discuss depression [246] Ask and talk about antidepressants [247] Unvoiced agenda [136] Communication behavior [248]
8. Decisions concerning treatment	1	Decisions concerning treatment	Decisions concerning treatment [96]
9. Discontinuation	2	Clinically advised discontinuation of a non-essential drug	Discontinuation [51,167]

**Fig 3:**
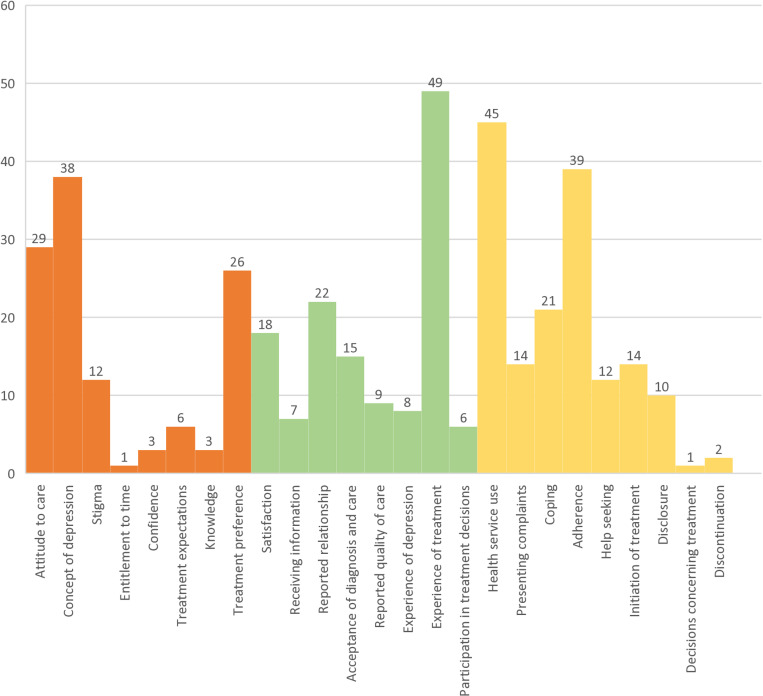
Patients’ perspectives on depression treatment in primary care: number of occurrences of topics and domains, i.e., ‘mindset’ (orange), ‘experience’ (green) and ‘behavior’ (yellow).

We identified 118 studies for ‘mindset’, 134 total occurrences within ‘experience’ and 158 occurrences within ‘behavior’. The most frequently occurring single topics were ‘experience of treatment’ (49), ‘health service use’ (45), ‘adherence’ (39), and ‘concept of depression’ (38). See also Fig 3.

Some topics, e.g., ‘preference’, were difficult to group as a whole because of heterogenous scientific approaches. While some studies asked for treatment preferences in more less informed patients, others involved their participants in shared decision making. Even other publications did not specify how informed their subjects were when asked for treatment preference or studied a heterogenous group in respect to that. When ‘discontinuation’ was studied, we made the distinction between discontinuation in terms of non-adherence to treatment (grouped as ‘adherence’) and clinically advised discontinuation of a non-essential drug (‘discontinuation’).

#### 4.5.2. Identified Barriers and Facilitators.

After establishing the framework, we rescreened all included publications to identify factors affecting, i.e., helping or impeding, patients’ behavior towards depression treatment in primary care. We identified factors inside and outside the established framework. Factors inside the framework are derived from either ‘mindset’ or ‘experience’.

**Fig 4:**
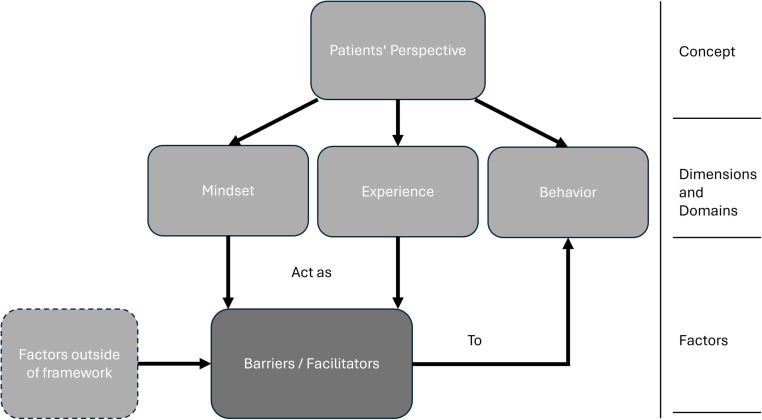
Framework and structure of ‘patients’ perspectives on depression treatment in primary care’ including barriers and facilitators.

Fig 4 gives an overview of the framework including barriers and facilitators as factors in context with the behaviors they relate to. In the model, barriers and facilitators are their own separate category consisting of factors from either ‘mindset’ or ‘experience’ on the one hand and influencing ‘behavior’ on the other hand. Factors outside the framework may also influence patients’ behavior.

Overall, we identified 61 publications testing and exploring barriers and facilitators to care. We decided on ‘help-seeking’, ‘disclosure’, ‘initiation’, ‘adherence’, and ‘discontinuation of medication’ as patient-sided relevant behaviors towards depression care. Most publications (n=39) dealt with ‘adherence’ and its barriers and facilitators. Only a few publications addressed help-seeking and maintenance of treatment. For an overview of all identified publications with all methodological approaches see Table 5. Qualitative studies with explorative approaches were more difficult to tag and group. We decided to label their topic as either ‘attitude to treatment’, if patients were naïve to treatment, or as ‘experience of treatment’, if patients had already started or undergone depression care in the past.

**Table 5:** Publications on barriers and facilitators to depression treatment in primary care from the patients’ point of view., including description of behavior and related factors inside the framework.

Behavior	Description	Factor - Mindset	Factor – Experience
Help-seeking	Time to initial medical presentation [241]	*Factors outside framework*	*Factors outside framework*
	Help-seeking patterns [88]	Stigma	Not being asked Experience of depression
	Help-seeking behaviors [44]	Attitude to treatment	Experience of treatment
	Help-seeking [45]	Attitude to treatment	Experience of treatment
	Help-seeking [46]	Attitude to treatment	–
	Not present in PC with depression [165]	–	Experience of treatment
	Help-seeking pathways [166]	–	Experience of treatment
	Uptake of GP referral [90]	Desire for emotional support Stigma of psychological help	–
	Intention to seek help [89]	Prejudice and discrimination Literacy for depression Perceived need for care	Self-identification as having a mental illness
Disclosure	Informing the physician of discontinuing antidepressants [30]	Attitude to drugs	Being told to stop by GP Relationship
	Symptom disclosure [244]	*Factors outside framework*	*Factors outside framework*
	Discuss depression [246]	*Factors outside framework*	*Factors outside framework*
	Disclosure [55]	Understanding postnatal depression	–
	Unvoiced agenda [172]	–	Relationship
	Not disclosing depression [48]	Fear of psychiatric or counselling referral Belief that depression is stigmatizing Medication aversion	Unsure how to introduce topic
	Disclosing symptoms [177]	–	Experience of disclosure
Initiation	Time to initial medical presentation [241]	*Factors outside framework*	*Factors outside framework*
	Perceived barriers to psychotherapy [95]	Concerns what others might think	Discomfort talking about personal issues with someone unknown
	Barriers to treatment and information [52]	Understanding of depression	Participation in treatment decisions Information given
	Filling prescription/ initiation of psychotherapy [41]	Stigma Beliefs about psychotherapy	–
	Initiation of and adherence to treatment (escitalopram or interpersonal therapy) [93]	Expectation Preference	–
	Participate in a depression treatment program [69]	Perceived cause of depression Perceived Stigma	Experience of seeking help
	Taking up referral to behavioral health [135]	–	Relationship
	Initiation of treatment [242]	*Factors outside framework*	*Factors outside framework*
	Initiation of treatment [243]	*Factors outside framework*	*Factors outside framework*
Adherence	Compliance to treatment (medication) [20]	Attitude to medication	Doctor-patient-communication
	Adherence to medication [130]	–	Being educated
	Adherence to treatment [131]	–	Quality rating Being educated Being empowered
	Adherence to medication [198]	*Factors outside framework*	*Factors outside framework*
	Compliance to antidepressants [30]	Attitude to drugs	Being told to stop by GP Relationship
	Adherence [57]	Illness model Controllability	–
	Why patients stop medication [155]	*Factors outside framework*	*Factors outside framework*
	Adherence to medication [122]	–	Satisfaction Experience of treatment
	Difficulty with continuation of care [149]	–	Quality of care Experience of treatment
	Adherence to maintenance pharmacotherapy [21]	Attitude to medication Confidence in managing side effects	–
	Adherence to medication [132]	–	Being educated
	Discontinuation [133]	–	Being educated
	Adherence [234]	*Factors outside framework*	*Factors outside framework*
	Adherence to sertraline [235]	*Factors outside framework*	*Factors outside framework*
	Medication concordance [38]	Beliefs about medication	Role of relationship Experience of medication
	Adherence to medication [24]	Drug attitude	–
	Treatment adherence [179]	–	Participation in treatment decisions
	Adherence to medication [28]	Attitudes to depression and its treatment	–
	Compliance to paroxetine [239]	*Factors outside framework*	*Factors outside framework*
	Adherence to medication [39]	Beliefs about medication	–
	Filling prescriptions [204]	*Factors outside framework*	*Factors outside framework*
	Compliance to antidepressants [148]	–	Agreement with treatment
	Completion of online-CBT [94]	Expectations of online CBT Experience of online CBT	–
	Initiation of and adherence to treatment (escitalopram or interpersonal therapy) [93]	Expectation Preference	–
	Initiation and continuation of antidepressant treatment [31]	Attitude to/ beliefs about drugs Health beliefs	Perceived norm (‘my GP thinks that I need to take medication’) Agreement with treatment
	Non-adherence [43]		*Factors outside framework*
	Adherence [166]	–	Experience of treatment
	Adherence [237]	*Factors outside framework*	*Factors outside framework*
	Adherence to medication [29]	*Factors outside framework*	*Factors outside framework*
	Care engagement [83]	Stigma Treatment preference	Agreement with depression label Report of provider behavior Received treatment
	Initiation of and adherence to Treatment [32]	Attitude to/ beliefs about drugs	Agreement with treatment
	Adherence to antidepressants [129]	–	Receiving information about antidepressants Experience of taking medication
	Discontinuation [240]	*Factors outside framework*	*Factors outside framework*
Discontinua-tion of medication	Discontinuation of antidepressants [167]	–	Experience of treatment
	Discontinuation of antidepressants [51]	Understanding depression Views on long term use of antidepressants	–

*: publications including qualitative data, **: publications limited to qualitative data

**Fig 5:**
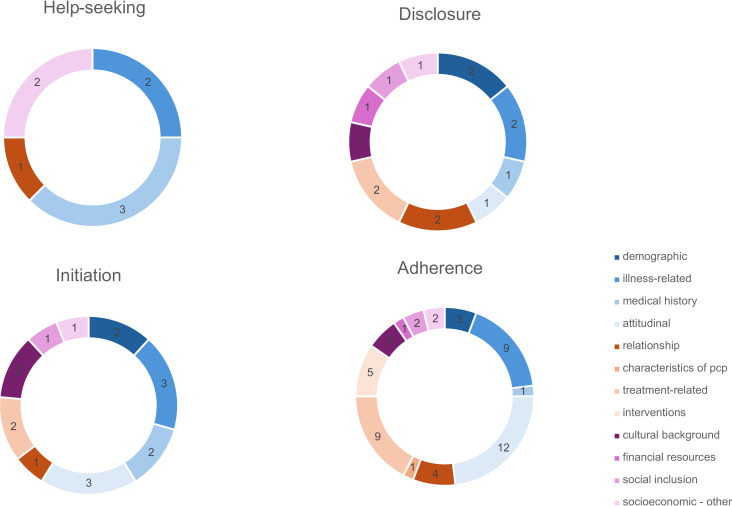
Number of publications containing barriers and facilitators, grouped as personal (blue shades), contextual (orange shades) and socioeconomic (purple shades).

Further we extracted, categorized, and grouped specific factors and synthetized them according to the framework. To link factors inside and outside the framework we established another way of grouping based on the data. We therefore assigned the extracted barriers and facilitators to the categories ‘personal’, ‘contextual’, and ‘socioeconomic’. These categories are based on several subcategories. See Fig 5. We defined ‘personal’ factors as all illness-related, attitudinal, and demographic aspects. Everything patients themselves bring to depression treatment in primary care.

‘Contextual’ is defined as all aspects of the doctor-patient-encounter that can act as barriers or facilitators. ‘Socioeconomic’ factors describe the social or financial status of patients with depression.

Table 6 gives details on factors within the framework, i.e., how mindset and experience act as barriers and facilitators to treatment-related patient behavior. Table 7 concludes findings that do not fit into our framework of patients’ perspective on depression treatment in primary care but were identified as factors of treatment behavior within the set of included publications.

**Table 6:** Patients’ mindset and experience acting as barriers and facilitators (factors) to patients’ behavior regarding depression treatment in primary care. Factors are divided into ‘personal’ (P), ‘contextual’ (C), and ‘socioeconomic’. Effects are given as significantly positively (+) or negatively (-) correlated with the behavior. ‘N.s.’ indicates that no significant influence was found for that factor.

	Domain	Description	Factor	Effect	Behavior	Source
Experience	Receiving information	Being educated	**C**	**+**	Adherence	Lin et al., 1995 Katon et al., 1995 Brook et al., 2005 Ruoff et al., 2005
	Relationship	Not being asked	**C**	**−**	Help-seeking	Van Hook, 1999
		Unsure how to introduce topic	**C**	**−**	Disclosure	Bell et al., 2011
		Better relationship	**C**	**+**		Demyttenaere et al., 2001
		‘Warm handoff’ (for Spanish-speaking Latinos)	**C**	**−**	Initiation	Horevitz et al., 2015
		Prescribed referral	**C**	**+**		
		Failure of communication	**C**	**−**	Adherence	Johnson, 1981
		Lower perceived norm (‘my GP thinks that I need to take medication’)	**C**	**−**		van Geffen et al., 2010
		Being empowered	**C**	**+**		Katon et al., 1995
	Acceptance of diagnosis and care	Less agreement with treatment	**P**	**−**	Help-seeking	Hérique, Kahn, 2009
		Self-identification	**P**	**+**		Schomerus et al., 2019
	Experience of treatment	Perceived separation between mental health and general health	**P**	**−**	Help-seeking	van Geffen et al., 2010
		Thinking that the staff is unhelpful and uninterested	**C**	**−**		Vuorilehto et al., 2016
		Being told to stop by GP	**C**	**−**	Adherence	Demyttenaere et al., 2001
	Experience of depression	Thinking that it did not affect their health	**P**	**−**	Help-seeking	Van Hook, 1999
	Participation in treatment decisions	More patient participation	**C**	**+**	Adherence	Loh et al., 2007
Mindset	Attitude to care	Belief that psychotherapy weakens the chance of obtaining a job	**P**	**n.s.**	Initiation	Stecker et al., 2007
		Medication aversion	**P**	**−**	Disclosure	Bell et al., 2011
		Attitude to/ beliefs about drugs	**P**	**−**	Adherence	Johnson, 1981 Demyttenaere et al., 2001 Weich et al., 2007 Russel, Kazantzis, 2008 van Geffen et al., 2010 Vuorilehto et al., 2016
		Attitude to drugs	**P**	**+**	Adherence	Lin et al., 2003 Brook et al., 2006 Russel, Kazantzis, 2008
	Concept of depression	Beliefs about depression	**P**	**−**	Adherence	Brown et al., 2001 Weich et al., 2007 van Geffen et al., 2010
		Beliefs about depression	**P**	**+**	Adherence	Weich et al., 2007
		Belief that one should be in control of depression	**P**	**−**	Disclosure	Bell et al., 2011
		[Depression] not doctor’s job	**P**	**−**		
		[Mood] private information	**P**	**−**		
	Stigma	Stigma	**P**	**−**	Help-seeking	Van Hook, 1999
		Stigma of psychological care	**P**	**+**		Holloway et al., 2015
		Blaming persons with mental illness for their problem	**P**	**−**	Help-seeking	Schomerus et al., 2019
		Belief that depression is stigmatizing	**P**	**−**	Disclosure	Bell et al., 2011
		Stigma	**P**	**n.s.**	Initiation	Stecker et al., 2007
		Internalized stigma	**P**	**−**	Adherence	Campbell et al., 2016
	Expectation	Fear of psychiatric or counselling referral	**C**	**−**	Disclosure	Bell et al., 2011
		Fear of being a ‘psychiatric patient’	**C**	**−**		
		Fear to distract doctor	**C**	**−**		
		Fear of being valued less	**C**	**−**		
		Fear of loss of emotional control	**C**	**−**		
		Medical records possibly unsafe	**C**	**−**		
		Concerns what others might think	**P**	**−**	Initiation	Mohr et al., 2006
	Confidence	Confidence in managing side effects	**P**	**+**	Adherence	Lin et al., 2003
	Knowledge	More knowledge	**P**	**+**	Help-seeking	Schomerus et al., 2019
	Preferences	Desire for emotional support	**P**	**+**		Holloway et al., 2015
		Discomfort talking about personal issues with someone unknown	**P**	**−**	Initiation	Mohr et al., 2006
		Strong preference for received treatment	**P**	**+**	Initiation	Raue et al., 2009
		Strong preference for received treatment	**P**	**+**	Adherence	Raue et al., 2009

**Table 7:** Barriers and facilitators outside of the framework of patients’ perspectives on depression treatment in primary care. Factors are divided into ‘personal’ (P), ‘contextual’ (C), and ‘socioeconomic’. Effects are given as significantly positively (+) or negatively (-) correlated with the behavior. ‘N.s.’ indicates that no significant influence was found for the factor.

	Factor	Description	Effect	Behavior	Source
Factors outside of framework	Personal	Female	–	Disclosure	Bell et al., 2011
		Female	+	Disclosure	O’Connor et al., 2001
		Female	–	Adherence	Katon et al., 2000 van Geffen et al., 2009 Kales et al., 2013
		Female	+	Adherence	Kales et al., 2013
		Male	–	Initiation	Cornwell et al., 2021
		Male	+	Initiation	Waitzfelder et al., 2018
		Senior patients	–	Initiation	Waitzfelder et al., 2018 Cornwell et al., 2021
		Age above 60 years	–	Adherence	van Geffen et al., 2009
		More Neuroticism	–	Help-seeking	Gormley et al., 1998
		More Neuroticism	–	Initiation	Gormley et al., 1998
		More severe depression	+	Help-seeking	van Hook et al., 1999
		More severe symptoms	–	Disclosure	Bell et al., 2011
		Higher depression scores	+	Disclosure	Bell et al., 2011 O’Connor et al., 2001
		More severe depression	+	Initiation	Waitzfelder et al., 2018
		Elevated depressive symptoms	–	Adherence	Vuorilehto et al., 2016
		More severe symptoms	+	Adherence	Brown et al., 2001
		Lower baseline PHQ-2	–	Initiation	Cornwell et al., 2021
		Less severe depression	–	Adherence	van Geffen et al., 2010
		Feeling better	–	Adherence	Demyttenaere et al., 2001 Manning et al., 2003 Hérique et al., 2009 Vuorilehto et al., 2016
		Fewer symptoms	+	Adherence	Russell et al., 2008
		Poor to moderate self-rated health	–	Adherence	van Geffen et al., 2009
		Unspecific symptoms	–	Adherence	van Geffen et al., 2010
		Longer illness	+	Adherence	Brook et al., 2006
		No history of depression	–	Initiation	Bell et al., 2011
		Prior mental health diagnosis	–	Initiation	Cornwell et al., 2021
		Fewer comorbidities	+	Initiation	Waitzfelder et al., 2018
		Previous mental health treatment	+	Initiation	Waitzfelder et al., 2018
		Coexisting personality disorder	–	Adherence	Akerblad et al., 2006
		Drug abuse	–	Adherence	Akerblad et al., 2006
	Contextual	Male physician	–	Adherence	Katon et al., 2000
		Full time physician	–	Adherence	Katon et al., 2000
		Previous contact with a psychiatrist	+	Disclosure	O’Connor et al., 2001
		Cost of psychotherapy	–	Initiation	Mohr et al., 2006
		Same day mental health appointment	+	Initiation	Cornwell et al., 2021
		High price of drugs	–	Adherence	Vuorilehto et al., 2016
		Shorter half-life of drugs	–	Adherence	Brook et al., 2006
		Number of prescribed medications	+	Adherence	Jeffery et al., 2023
		Side effects	–	Adherence	Johnson, 1981 Demyttenaere et al., 2001 Brook et al., 2006 Dernovsek et al., 2008 Hérique et al., 2009 Vuorilehto et al., 2016
		Occurrence of adverse effects	+	Adherence	Brook et al., 2006
		Lack of efficacy	–	Adherence	Demyttenaere et al., 2001 Hérique et al., 2009 Vuorilehto et al., 2016
		Treatment worked quickly	–	Adherence	Manning et al., 2003
		Early treatment response	+	Adherence	Brook et al., 2006
		Good effect of medication	+	Adherence	Serrano et al., 2014
		Prior visit to clinic	–	Initiation	Cornwell et al., 2021
		Telemedicine-based collaborative care	+	Adherence	Fortney et al., 2011
	Socioeconomic	Hispanic	–	Disclosure	Bell et al., 2011
		Asian, African American or Hispanic	–	Initiation	Waitzfelder et al., 2018
		Hispanic	+	Initiation	Cornwell et al., 2021
		Non-western immigrants	–	Adherence	van Geffen et al., 2009
		African American	–	Adherence	Kales et al., 2013

For the synthesis in Table 6 and 7 we only included quantitative results from mentioned publications. To comprehensively extract qualitative findings conducting a detailed qualitative meta-synthesis would have been needed.

We identified quantitatively researched factors affecting patients’ behavior that can either be attributed to patients’ mindset or experience, i.e., related to our framework, in 24 of the publications. Quantitatively researched factors outside the framework derived from 24 publications.

Overall, most barriers and facilitators were grouped as either illness- (i.e., personal) or treatment-related (i.e., contextual) or attitudinal (i.e., personal). Socioeconomic factors, the impact of practitioners’ characteristics and the doctor-patient relationship were less researched. See Fig 5 for an overview of the distribution of all factors.

For help-seeking we extracted specific findings from four publications. These findings focused especially on the importance of internalized and externalized stigma as a barrier, while knowledge and more severe symptoms seem to be enabling. Proactively asking for symptoms on the practitioners’ side (i.e., ‘being asked’) showed to be important when thinking about seeking help.

Three publications gave quantitative data on barriers and facilitators to disclosure. Interestingly different studies showed contradictory findings on whether female gender leads to more or to less disclosure. The same applied for symptom severity, where two studies found more severe symptoms to be an enabler, one study found them to be hindering. Again, attitudinal factors related to stigmatizing beliefs about the disorder, the treatment, and the context, showed to be effective barriers. The quality of patient-doctor-relationship was studied as an effective facilitator of disclosure. Two studies found socioeconomic barriers such as low educational and financial status as well as ethnic identity.

We extracted quantitative findings from eight publications tackling barriers and facilitators towards the initiation of treatment. Again, there were contradicting findings regarding gender as well as ethnic background. While stigmatizing beliefs showed to be hindering in one study, another study could not show stigma to be a barrier. Consistently, high depression severity was related to a lower threshold to disclosure. For Hispanics, formal referrals showed better rates of initiation compared to informal referrals (‘warm handoff’). Experience with treatment as well as preference also showed to have an impact on disclosure. Financial and time concerns are a barrier treatment initiation, which can be helped by offering timely appointments.

Adherence constitutes the most studied patient-sided aspect of depression care as it is regularly assessed in drug trials. Overall, we included 27 studies offering quantitative data on adherence and its barriers and facilitators. Treatment- and disease-related factors seemed important in prematurely discontinuing antidepressants, i.e., feeling better or not registering an effect, low symptomatic burden, and side effects. Also, attitudes and beliefs towards drugs show an impact on adherence. Educating patients has shown to be a facilitator of effective treatment with antidepressants.

#### 4.5.3. *Presenting Complaints.*

We took a closer look at the presenting complaints or symptoms that patients reported when depression is present or diagnosed. While psychological complaints such as ‘low mood’ point practitioners into the right direction, the primary presentation of physical symptoms might on the other hand impede diagnosis. Even though most publications considered in this section alluded to this effect, they did not examine its actual impact on treatment outcomes, i.e., if it is an actual barrier in the sense of our definition. Presenting complaints itself is a behavior but could be indicative of mindset, e.g., beliefs about illness. Also, symptom report can be more easily acquired and looked at in a clinical or research context as for instance attitudes and beliefs themselves.

We included all publications on ‘presenting complaints’ with the exception of two [218,220] which did not report the relevant data in a manner that appeared useful for the synthesis. Tylee et al. studied number and timing symptom report within the consultation [223]. We included the publication in the following table for an overview over the methodology without extracting the ‘percentage of visits for somatic complaints.

Due to our inclusion criteria, all outcomes for this category derived from open-ended questions. The reported symptoms were further categorized by the authors. We defined ‘somatic complaints’ as physical, vegetative, or somatic symptoms. Table 8 provides an overview of included publications and extracted data.

**Table 8:** Publications covering presenting complaints.

Publication, First author and year	Outcome	Number of Participants	Results	Percentage of visits for somatic complaints
Widmer et al., 1978	Chief complaint	154	At time of diagnosis 23% of depressed patients gave low mood, 33% sleep disturbances, 31% fatigue, 4% anxiousness or agitation and 9% other symptoms as their chief complaint.	64
Diamond et al., 1987	Reason for visit	67	The reasons for visit given by patients were 34.5% somatic complaints, 19.5% vegetative and 21% psychological.	60
Williamson et al., 1989	Initial complaint	7	In the family practice center depressed patients gave following initial complaints: “General yuck”, Physical exam, Kidney problem, Injured finger, Severe headaches, “Multiple complaints”, Arthritis.	100
Kirmayer et al., 1993	Presenting complaints	202	Of the patients with CES-D scores of 16 or higher, 14% (N=31) were psychosocial presenters, 32% (N=69) were initial somatizers, 24% (N=52) were facultative somatizers, and 23% (N=50) were true somatizers. The presentations of 6% (N=13) were classified as health maintenance visits. These patients were excluded from further comparisons. Facultative and true somatizers only reported physical complaints.	85*/50
Tylee et al., 1995	Mentioning of symptoms	72 (women)	A total of 13 mentioned between 10 and 19 physical symptoms and four patients mentioned between 20 and 29 physical symptoms. The first mention of a psychiatric symptom was within the first four mentions of any symptoms for 30 women. 41 women first mentioned a psychiatric symptom after the first four mentions or mentioned no psychiatric symptom.	N/A
Cornwell et al., 1998	Presenting complaints	90 (40 South Asian, 50 White persons)	Within the South Asian group 27 (67,5%) presented with physical complaints, 38 (95%) presented with psychological complaints (n=40). In the White group 11 (22%) presented with physical complaints, 50 (100%) presented with psychological complaints (n=50).	68*/ 22*
Waza et al., 1999	Symptom report	189 (85 USA, 104 Japan)	USA (N=85): 74.1% also reported physical symptoms, 9.4% reported only physical symptoms, 16.5% reported only psychological symptoms. Japan(n=104): 69.3% also reported physical symptoms, 26.9% reported only physical symptoms, 3.8% reported only psychological symptoms.	74*/ 69*
Mckelvey et al., 2001	Presenting complaints	1057	Of the patients with a CES-D above the cut-off 77% (813) presented with physical complaints, 23% (244) presented with psychological complaints.	77
Yeung et al., 2004	Presenting complaints	29	The most common presenting complaints were fatigue (N=5), insomnia (N=5), headache (N=4), cough (N=2), pain (N=2), dizziness (N=2), cervical problems (N=1), and sexual dysfunction (N=1). Four complained of psychological symptoms, 2 described feelings of nervousness. One did not spontaneously report any symptoms.	76
Menchetti et al., 2009	Reason for consultation	250	110 depressed patients (44.0%) consulted their PCP for psychological or family problems, 102 subjects (40.8%) for physical illness, and 38 subjects (15.2%) for pain symptoms.	56
Chen et al., 2015	Chief complaint	190	Subjects were most likely to report a chief complaint in the category of Depressed mood/ unhappiness/mood problems (52.1%), followed by Psychosocial stressors (38.9%), Depressive neurovegetative symptoms (31.6% — e.g., poor sleep, poor appetite, low energy), and Depressive psychological symptoms (24.2% — e.g., rumination, difficulty concentrating, agitation). Multiple responses were allowed.	32*
Heinz et al., 2021	Reporting complaints	430	190 (44.2%) patients with depression reported physical complaints only, 42 (9.8%) reported mental complaints only, 124 (28.8%) consulted the GP due to mental and physical complaints, 19 (4,4%) stated no complaints and 55 (12.8%) gave other reasons.	44/ 29*

* Percentage of participants also, not necessarily exclusively, reporting physical complaints.

Between 44–100% of studied patients presented only with physical symptoms. The percentage of patients also reporting physical symptoms when seeking help for depression was given between 22 and 85%. Some publications also studied the correlation between depression state and number of symptoms given, which we did not further synthesize [87,223].

## 5. Discussion

### 5.1. Overall findings

With this review we established and defined the concept of ‘patients’ perspectives on depression treatment in primary care’ based on available research, identified and structured relevant research terms as well as methodological approaches. Patients’ mindset and experience can either hinder or help with their engagement in treatment, thus serving as barriers or facilitators (factors) to certain treatment-related behaviors.

Different personal, socioeconomic and contextual factors have been researched in their role as barriers and facilitators. Promising target points for intervention for practitioners are educating patients, addressing attitudes and preferences as well as proactively asking for depression symptoms.

When seeking help for depression in primary care many patients exclusively or also mention physical symptoms, which can impede finding the right diagnosis and receiving adequate and timely treatment.

Even though depression is very common and often treated exclusively in the primary care context, still most research towards depression care is conducted in specialized contexts [249]. Looking at the patients’ perspective of care beyond symptoms is sometimes a secondary topic, when conducting a randomized-controlled-trial, but has also been a researched outcome since at least the 1970s. The majority of publications related to our research questions were published in the early 2000s. Including patients as important stakeholders not just as subjects but also in planning and conducting research has been a growing trend in recent years [250]. It is to be expected that this will lead to more publications on the patient’s perspective in years to come.

Even though we limited our findings to OECD member states for the screening process it is still notable that most publications stemmed from the Anglo-American region. Our research zooms in on a very distinct context that depends on not only individual but also social and political parameters. We cannot say if findings from the US apply for German primary care settings or vice versa. On the other hand, primary care itself and its research is a heterogenous field with a multitude of settings and scientific approaches.

Based on the literature we provide a comprehensive framework for the concept ‘patients’ perspective on depression treatment in primary care’ which can be used for future studies, applied internationally, and be built on creating networks and foci for research and intervention. The implementation of the patients’ perspectives in future guidelines and policy is essential to work towards more accessible care for all patients with depression. The primary care context provides an ideal setting for impactful research and intervention [251]. In terms of methodology, we noted that less than 60% of included publications used comparable measures to identify depressed patients such as diagnostic interviews and questionnaires hindering more in-depth data synthesis.

### 5.2. Patients’ perspectives on depression treatment in primary care

With this work we provide an overview of literature on patients’ perspectives on depression treatment in primary care. We defined and evaluated a structured approach to the concept trying to grasp its width and depth. By evaluating the method throughout the entire process, we ensured an objective and understandable approach to literature synthesis.

We identified and grouped topics we consider relevant to the concept of patients’ perspective. The premeditated dimension seemed useful for that process.

Comparing domains, topics, and their numbers of occurrences, we found that research approaches and scientific research towards them varies. Behavioral topics have been studied since at least the 1970s and provide clear comparable quantitative measures. The fact that patients’ behavior is observable and close to a physician-centered approach is a possible explanation.

Apart from behavioral topics we found that ‘satisfaction’ and ‘preference’ are commonly studied outcomes. They rarely constitute main outcomes of studies but are easily retrievable data derived from longitudinal and cross-sectional studies. After quality appraisal these publications can be included in separate systematic reviews. Based on our review, we recommend that these outcomes should be included in future intervention studies. We also recommend precisely stating how these outcomes were generated to provide accessible and comparable data for evidence synthesis and future research.

Surprisingly, we only identified four publications directly researching patients’ expectations [36,56,93,94]. Even though many terms grouped under ‘mindset’ are related to ‘expectation’ it is rarely directly addressed in studies. This constitutes an important research gap as expectation has shown to be a meaningful mediator of many clinical outcomes [252].

Topics like ‘acceptability’, ‘preference’ and ‘satisfaction’ are possibly dependent on expectations and could be predicted by them. Further research could close that gap and lead to an early intervention instead of hindsight, as measured by ‘satisfaction’. Current research stresses the link between patient-provider-relationship and satisfaction [253].

The terms ‘experience of depression’ and ‘experience of treatment’ are broad and more complex to be synthesized. Publications on those terms used a qualitative approach. We did not perform a qualitative metasynthesis, which would be an interesting next step regarding these topics.

### 5.3. Barriers and facilitators to treatment faced by patients

According to our definition of ‘barriers and facilitators’ i.e., conditions and interventions impeding and helping with care, we identified and grouped related topics. The concept of ‘barriers and facilitators’ as factors by our definition linked all three dimensions as shown in Fig 4. Both mindset and experience influence treatment related behavior. For instance, certain topics we categorized as ‘mindset’ were also identified as factors affecting treatment, e.g., ‘stigma’ and ‘attitude to care’.

For scoping, we found this framework to be adequately clear and inclusive.

Researched factors of help-seeking, disclosure, initiation, adherence, and discontinuation varied in terms of number of publications and outcomes. For the diagnostic stage of treatment, i.e., help-seeking and disclosure, stigmatizing beliefs seem to be an important barrier. For the actual treatment, i.e. initiation and adherence, symptom burden attitudes towards and knowledge of treatment and illness play an important role.

Most research focused on adherence and lack thereof, often contributing as a secondary outcome to large-scaled medication studies. While drug-associated barriers like side effects, level and course of symptom burden may not be specific to the primary care setting, barriers and facilitators describing the patient-provider-relationship may be. This has been shown for other medical issues and drugs [254]. Interventions involving educating patients have shown to be effective, which may also deal with commonly found barriers stemming from beliefs and attitudes towards drugs.

Preference for the prescribed treatment [93] and participation [179] have also been described as helpful to improve patients’ motivation for and therefore adherence to care. Nevertheless, more research is needed to build from these findings.

We did not find research on geographic and other community factors as factors of help-seeking and care, even though we believe it to have a big impact especially when it comes to under-served communities, who might be underrepresented in current research [255]. Among our inclusions, socioeconomic factors are less researched than personal and contextual factors. Sex and ethnicity were often researched as barrier or facilitator while findings are contradictory, a possible explanation being that these categories are oversimplified and highly influenced by other factors.

Overall, factors that were not directly related to treatment and illness, e.g., attitudinal or relationship aspects, were less researched. With the rise of integrated concepts like collaborative care [256], we believe these aspects as important to be included into future research and policy.

Among our included publications there are also other topics arising that are related to barriers and facilitators to care even though they do not clearly frame it that way. Not just when patients disclose symptoms but also the type of presenting complaints influences if they are diagnosed correctly. Among publications studying the quality and quantity of complaints there is a trend towards patients reporting physical symptoms. Three of the identified studies correlated the quality (physical vs. psychological), quantity and timing of complaints with the correct recognition of the depressive state by the GP and found that the report of physical symptoms as well as the delayed report of psychological symptoms reduce the chances of being diagnosed with depression [206,222,223]. Regarding our framework, help- seeking can be seen as a both quantitative, whether or not they seek help, and qualitative, for what they seek help, i.e., disclosing psychological symptoms (‘disclosure’/’symptom report’) or reporting mainly physical symptoms of depression (‘symptom report’).

Reporting mainly physical complaints might also be a contextual effect. Williamson et al. compared patients visiting a family practice with those consulting a psychiatric clinic and found this to be a distinction, i.e., primary care patients reporting more somatic symptoms [217]. Other contextual effects such as sociocultural aspects, national and regional, might also play a role on patients’ symptom report [18]. It is important to further research presenting complaints and behaviors with the questions whether and to what degree they can impede proper diagnosis.

Data about patient-sided barriers and facilitators towards depression treatment in primary care still seems inconclusive with contradictory and heterogenous approaches and results. More research and synthesis are needed for which we provided a framework.

### 5.4. Strengths and limitations

To our knowledge, this is the first literature review on patients’ perspectives on depression treatment in primary care offering a comprehensive framework for future studies.

We conducted a premeditated and thorough search for all relevant studies. Because the findings were limited to the primary care context, we naturally excluded other designs and studies that may have contributed to the question of patients’ perspectives, especially mindsets.

We limited our search to MEDLINE and Psycinfo, not specifically looking for grey literature. We believe our data to be representative of current research and our framework to be applicable for possibly missed publications. Because we wanted our framework to be inclusive, we included publications without time limitations. Possibly we did not find and include older studies beyond the 1970s because the term ‘depression’ was less common. Older studies probably would have differed greatly in terminology and concept of depression and would have been difficult to compare to newer studies. Included older publications, especially from the 1980s, contributed to our framework as they often focused on health service use or symptom report. Research on these topics has since declined.

We think that the identified research topics point out important factors for treatment. We conceptualized the abstract term ‘perspective’ for the purpose of our research and found it sufficiently broad and specific. We do not claim our concept of ‘perspective’ to be the only valid one. We encourage future investigators to critique and build from our concept.

In the planning stage of this project, we were advised on its design by patients from the *Munich Alliance Against Depression* (Münchner Bündnis gegen Depression e.V.) an interest group organized in Germany for patients with a history of depression.

## 6. Conclusion

This scoping review defines ‘patients’ perspective on depression treatment in primary care’ as a scientific field with its domains, topics, and research approaches and provides a comprehensive overview of available literature, and providing a framework for future studies. Apart from ‘adherence’ relevant topics lack comparable data to draw solid conclusions from. This is partly due to heterogenous approaches to how depression is diagnosed. Apart from quantitative data, a lot of potential lies in qualitative approaches and their metasynthesis, e.g., for the topic of ‘patients’ experience. Patients’ behavioral measures are a straightforward way to implement patients’ perspectives as a secondary outcome in prospective and cross-sectional study designs.

Findings about patient-sided factors influencing care which are already described and studied are summarized as well as grouped in an approachable and systematic manner to help guide future research. While illness- and treatment-related barriers and facilitators are commonly researched, there is less data on how the patient-provider-relationship impacts patient behavior. While adherence is focused on most commonly, there is little evidence on what hinders or encourages help-seeking, disclosure, or maintenance. Educating patients has shown to be enabling patients’ adherence to treatment. Sex and ethnicity seem to have an impact, even though still ambivalent. Possible mediating factors are attitude and beliefs towards illness and treatment. Stigma has shown to be hindering help-seeking and disclosure. Patients often present with physical symptoms when seeking help for depression which can hinder or delay diagnosis.

Patients’ perspectives on depression treatment in primary care are an essential aspect of the maintenance and improvement of evidence-based care and should be included in guidelines and policies.

## 7. Clinical implications

Even though there is a trend towards more patient participation in research [257], clinical practice guidelines still rarely consult patients with their experiences in the process of making decisions on recommendations and implementation. When it comes to depression treatment in primary care the lack of guideline-implementation possibly leads to less strategic and thus less effective care. Apart from contextual barriers, patient-sided factors also seem important to consider. With this review we offer an evidence-based framework for this concept, especially focused on barriers and facilitators to partaking in depression care.

The purpose of this framework is three-fold: First, it can be used as a base for questionnaires researching the concept of patients’ perspectives on depression treatment in primary care. Quantifiable measures, such as satisfaction, adherence, or preference, are easy to integrate into clinical trials and offer the possibility for comparison and meta-analysis.

Second, patient-related measures about their experience and expectations can help to guide policies, to validate and evaluate models of quality of care, to improve guideline development processes, and consequently can make treatment more patient-centered, targeted and efficient. Effective implementation of guidelines and changes in heuristics in primary care require research that includes perspectives of all relevant stakeholders along with contextual factors. To create and test tailored implementation strategies it is crucial to comprehensively understand contextual barriers and facilitators [258]. A better understanding of help-seeking and facilitating interventions seems promising to help with early diagnosis in primary care, prevent chronicity, and reduce the burden of depression.

Third, the synthesis of barriers and facilitators offers a theoretical base on which networks can be built to better understand what the possible targets for intervention are. An understanding of contextual barriers from the patients’ point of view can help to tailor effective implementation strategies. Educating patients and addressing stigmatizing beliefs are promising targets to promote the seeking out, initiation of, and adherence to treatment.

**Fig 6:**
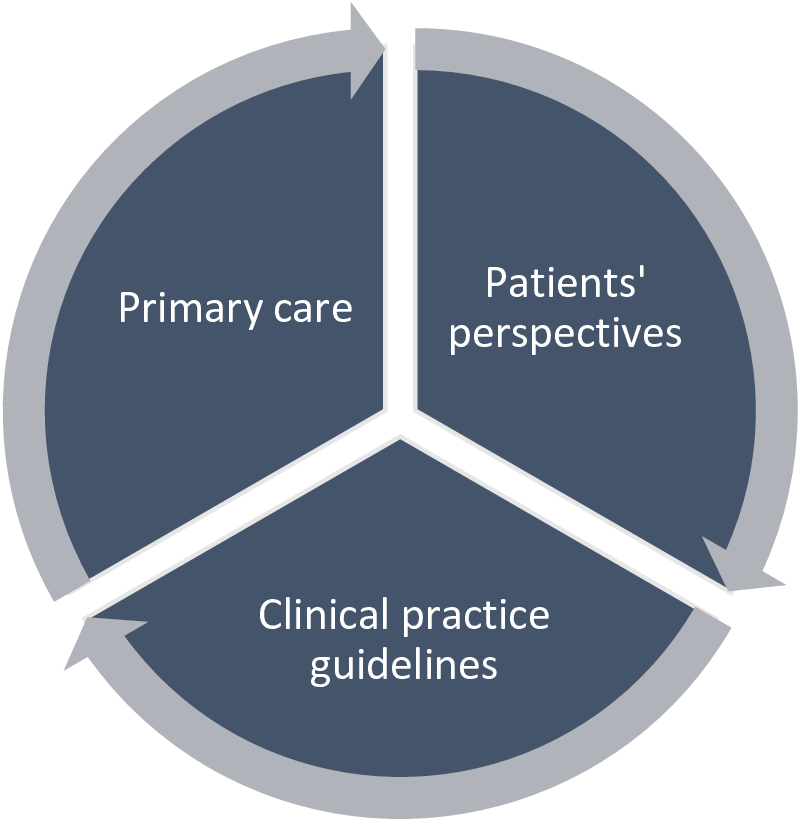
Model of impact of patients’ perspectives on clinical practice guidelines, primary care, and vice-versa.

Fig 6 shows a theoretical model of how patients’ perspectives on care can be included into the forming and implementation of clinical practice guidelines to help with evidence-based and effective treatment of depression in primary care.

We have shown that the concept of ‘patients’ perspectives on depression treatment in primary care’ is both complex and important for successful treatment strategies.

## Supporting information

S1 Appendix Appendix 1: Mesh terms and iterations. Appendix 2: Search strategy. Appendix 3: Included publications and extracted data. (DOCX)

S2 Checklist (DOCX)
